# Molecular characterization and functional annotation of a hypothetical protein (SCO0618) of *Streptomyces coelicolor* A3(2)

**DOI:** 10.5808/GI.2020.18.3.e28

**Published:** 2020-09-21

**Authors:** Nadim Ferdous, Mahjerin Nasrin Reza, Md. Tabassum Hossain Emon, Md. Shariful Islam, A. K. M. Mohiuddin, Mohammad Uzzal Hossain

**Affiliations:** 1 Department of Biotechnology and Genetic Engineering, Faculty of Life Science, Mawlana Bhashani Science and Technology University, Tangail 1902, Bangladesh; 2 Laboratory of Reproductive and Developmental Biology, Hokkaido University, Sapporo 060-0808, Japan; 3 Bioinformatics Division, National Institute of Biotechnology, Savar, Dhaka 1349, Bangladesh

**Keywords:** *Streptomyces coelicolor*, genome, hypothetical protein, modeling, hydrolases

## Abstract

*Streptomyces coelicolor* is a gram-positive soil bacterium which is well known for the production of several antibiotics used in various biotechnological applications. But numerous proteins from its genome are considered hypothetical proteins. Therefore, the present study aimed to reveal the functions of a hypothetical protein from the genome of *S. coelicolor*. Several bioinformatics tools were employed to predict the structure and function of this protein. Sequence similarity was searched through the available bioinformatics databases to find out the homologous protein. The secondary and tertiary structure were predicted and further validated with quality assessment tools. Furthermore, the active site and the interacting proteins were also explored with the utilization of CASTp and STRING server. The hypothetical protein showed the important biological activity having with two functional domain including POD-like_MBL-fold and rhodanese homology domain. The functional annotation exposed that the selected hypothetical protein could show the hydrolase activity. Furthermore, protein-protein interactions of selected hypothetical protein revealed several functional partners those have the significant role for the bacterial survival. At last, the current study depicts that the annotated hypothetical protein is linked with hydrolase activity which might be of great interest to the further research in bacterial genetics.

## Introduction

*Streptomyces coelicolor* A3(2) is one of the best studied representatives amongst other members of the genus Streptomyces [1]. Like the streptomyces genus in general, it lives in soil [2]. It is considered a model organism to study soil bacteria [3], which has been studied genetically for about 60 years [4]. They have the capability to degrade chitin and other compounds that are difficult to degrade which makes them especially important [5]. This bacterium produces a range of secondary metabolites, including actinorhodin, undecylprodigiosin, calcium-dependent antibiotic, methylenomycin A and perimycin [6]. Some of them have antifungal activities also. So, *Streptomyces coelicolor* A3(2) has the potential to make such secondary metabolites, and metagenomic analysis has revealed it has tremendous quantities of significant biosynthetic gene sets [7,8].

These characteristics have elicited biotechnological interest in this bacterium and have aroused the interest of researchers in the past few years to investigate the different proteins involved in secondary metabolites production. As an example, it is recently found that albaflavenone, germicidin A, and chalcone are produced during germination of *Streptomyces coelicolor* [9] and the genes responsible for the biosynthesis of streptomycete secondary metabolites are generally clustered with high expression of regulation [10]. Another research shows that a group of mtbH-like genes in *S. coelicolor* are necessary for some secondary metabolite production [11]. *Streptomyces coelicolor* has three such genes, cloY is one of them [11]. When all three genes were absent, clorobiocin, an antibiotic which inhibits the enzyme DNA gyrase was produced only in very small amounts, but when cloY was restored, clorobiocin was produced at a more significant level [11].

*Streptomyces coelicolor* A3(2) is reported to have 8,667,507 base pair linear chromosome, containing the largest number of genes so far discovered in a bacterium [10]. The genes so far predicted are 7,825 which include more than 20 clusters coding for known or predicted secondary metabolites [10]. However, there are many proteins of this bacterium which are considered hypothetical proteins as their structures and biological functions are not yet known. These proteins can be very important and their annotation can lead to knowledge about new structures, pathways, and functions. Thus, bioinformatics approaches can play an important role in predicting and analyzing various forms of structure of those hypothetical proteins, their biological functions as well as protein-protein interactions.

With the advancement of in-silico analysis, it became easier to annotate function to a hypothetical protein using various bioinformatic tools. Thus, the purpose of this study was to assign structural and biological function to the hypothetical protein SCO0618 (accession No. NP_624929.1) of *S. coelicolor* for an improved understanding of the protein. Subcellular localization, secondary structure, and active site were predicted and protein-protein interaction was analyzed. Further, a good quality model of the SCO0618 was tried to generate using homology modeling techniques.

## Methods

### Sequence retrieval and similarity identification

The sequence information of the hypothetical protein (NP_624929.1) was retrieved from the NCBI database. The sequence was then collected as a FASTA format sequence and submitted to several prediction servers for the in-silico characterization (Table 1). To get the initial prediction about the function of the targeted hypothetical protein, similarity search was performed with the NCBI protein Database (https://www.ncbi.nlm.nih.gov/) against nonredundant and SwissProt [12] database to find out the proteins that might have structural similarities with that of the uncharacterized protein by using BLASTp program [13].

### Multiple sequence alignment and phylogeny analysis

Multiple sequence alignment was performed using MUSCLE server of EBI (https://www.ebi.ac.uk/Tools/msa/muscle/) [14] and visualized using the CLC Sequence Viewer 7.0.2 (http://www.clcbio.com). The phylogeny analysis was done by using the webtool Phylogeny.fr (http://phylogeny.lirmm.fr/) [15].

### Physiochemical properties analysis

The physical and chemical properties including molecular weight, theoretical pI, amino acid composition, atomic composition, extinction coefficient, estimated half-life, total number of negatively charged residues (Asp + Glu), total number of positively charged residues (Arg + Lys), instability index, aliphatic index, and grand average of hydropathicity (GRAVY) predictions, etc. were performed by the ProtParam (http://web.expasy.org/protparam/) [16] tool of ExPASy.

### Subcellular localization analysis

Subcellular localization was predicted by CELLO [17]. Results were also cross-checked with subcellular localization predictions obtained from PSORTb [18], PSLpred [19], and SOSUIGramN [20]. TMHMM [21], HMMTOP [22], and CCTOP [23] were used for the topology prediction.

### Conserved domain, motif, fold, coil, family, and superfamily identification

Search carried out at conserved domain database (CDD, available at NCBI) [24], for conserved domain. Protein motif search was carried out using Motif (Genome Net) server [25]. Pfam [26] and SuperFamily [27] database searches were done to assign the protein’s evolutionary relationships. For the detection of coiled-coil conformation within the protein, the COILS server [28] was employed. Protein sequence analysis and classification server InterProScan [29] was employed for the functional analysis of the protein. For protein folding pattern recognition, PFP-FunD SeqE server [30] was used. And STRING 10.0 [31] search was carried out for the identification of possible functional interaction network of the protein.

### Secondary structure prediction

PSI-blast based secondary structure Prediction (PSIPRED) [32] and self-optimized prediction method with alignment (SOPMA) servers were used for the prediction of the proteins’ secondary structure [33].

### Three-dimensional structure prediction

The three-dimensional structure was predicted by HHpred server (https://toolkit.tuebingen.mpg.de/tools/hhpred) [34] of the Max Planck Institute for Developmental Biology, Tübingen which is based on the pairwise comparison profile of hidden Markov models (HMMs). For higher accuracy, the 3D structure was predicted on the basis of best scoring template. Later the 3D structure was refined through YASARA energy minimization server [35].

### Model quality assessment

Finally, PROCHECK (https://servicesn.mbi.ucla.edu/PROCHECK/) [36], Verify3D (http://nihserver.mbi.ucla.edu/Verify_3D/) [37], and ERRAT Structure Evaluation server (https://servicesn.mbi.ucla.edu/ERRAT/) [38] were used for quality assessment of the predicted three dimensional structure.

### Active site detection

The active site of the protein was determined by the Computed Atlas of Surface Topography of Protein (CASTp) (http://sts.bioengr.uic.edu/castp/) [39] which provides an online resource for locating, delineating, and measuring concave surface regions on three-dimensional structures of proteins.

## Results and Discussion

**Fig. 1.**
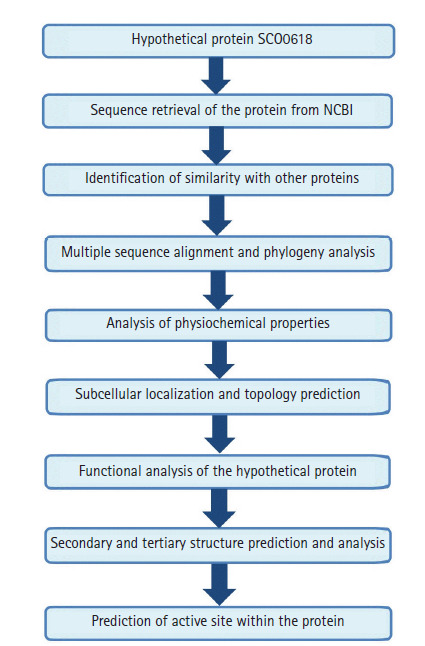
A complete workflow of the study.

The work-flow of the study was shown in Fig. 1.

### Sequence and similarity information

**Fig. 2.**
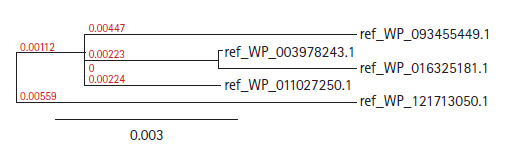
Phylogenic trees with true distance of different hydrolases proteins.

The Blastp result against non-redundant and SwissProt database showed homology with other hydrolase and sulfurtransferase proteins (Tables 2 and 3). Multiple sequence alignment (Supplementary Fig. 1) was considered the FASTA sequences of the hypothetical protein (SCO0618) and the homologous annotated proteins. For the confirmation of homology assessment between the proteins, down to the complex and subunit level, phylogenetic analysis was also performed. Phylogenetic tree was constructed based on the alignment and BLAST result which gives the similar concept about the protein (Fig. 2). The distances between branches are also included.

### Physicochemical features

The protein consist of 461 amino acids, among the most abundant were Ala (92) followed by Val (51), Arg (42), Gly (41), Leu (40), Asp (32), Glu (30), Pro (26), Thr (21), Ser (19), His (17), Phe (11), Ile (10), Tyr (8), Trp (6), Asn (5), Gln (4), Met (4), and Cys (2). The calculated molecular weight was 48216.15 Da and theoretical pI was 5.27 indicating the protein to be negatively charged. Total number of positively charged residues (Arg + Lys) and the total number of negatively charged residues (Asp + Glu) were found to be 62 and 42, respectively. The computed instability index was 32.67 classifying the protein as stable one. Aliphatic index was 94.34 which gives an indication of proteins’ stability over a wide temperature range. The grand average of hydropathicity (GRAVY) was 0.053. Positive value of GRAVY indicates that the protein is polar. Protein half-life computed was found to be 30 h in mammalian reticulocytes (in vitro), >20 hours in yeast (in vivo), >10 h in *Escherichia coli* (in vivo). And the molecular formula of protein was identified as C_2119_H_3350_N_636_O_643_S_6_.

### Functional annotation of the hypothetical protein

**Fig. 3.**
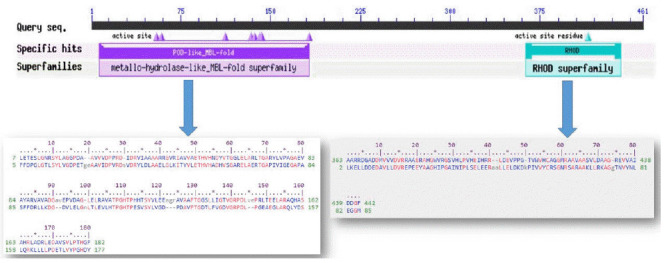
Functional annotation of the hypothetical protein.

**Fig. 4.**
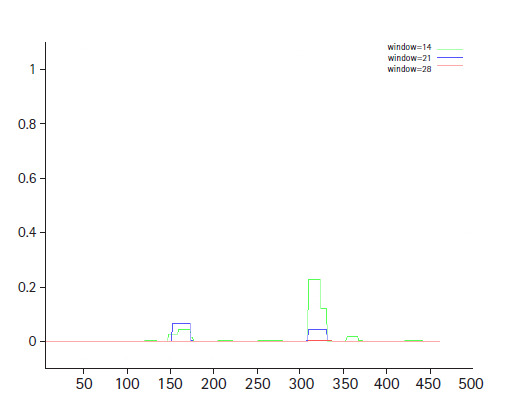
Coil depicts the heptads corresponding to the residue windows 14 (green), 21(blue), and 28 (red).

The conserved domain search tool revealed that this hypothetical protein sequence was found to have two domains, MBL-fold metallo-hydrolase domain (accession No. cd07724) and rhodanese homology domain (RHOD) (accession No. cd00158). The result was also checked by two other domain searching tools namely InterProScan and Pfam. Pfam server predicted the Rhodanese like domain at 362–444 amino acid residues with an e-value of 2.3e-05 and Metallo-beta-lactamase superfamily domain at 16–171 amino acid residues with an e-value of 4.7e-07. InterproScan server predicted Rhodanese like domain at 249–454 amino acid residues and Metallo-beta-lactamas domain at 13–180 amino acid residues. Rhodanese like domain, lactamase-B and MreB-Mbl domains were also found by Motif server. Superfamily search revealed present of Metallo-hydrolase/oxidoreductase and rhodanese/cell cycle control phosphatase superfamily. β-Lactamases can catalyze the hydrolysis of a wide range of β-lactam antibiotics. Members of the MBL-fold metallohydrolase superfamily are mainly hydrolytic enzymes which carry out various biological functions. Both the active and inactive version of the Rhodanese domain in a variety of proteins including certain protein phosphatases, sulfide dehydrogenases, certain stress proteins and sulfuryl transferases, where they are thought to play a regulatory role in multidomain proteins (Fig. 3). All these results confirm the presence of hydrolytic enzyme containing domains in this protein. Fold pattern recognition by PFP-FunDSeqE tool revealed the presence of a ‘(TIM)-barrel’ fold within the protein sequence. (TIM)-barrel structure is generally eight stranded α/β barrel. The x-axis of the graph represents the position in the protein of amino acid number (starting at the N-terminus) and the y-axis shows the coiled coil whereas ‘Window’ refers to the width of the amino acid ‘window’ that is scanned at one time (Fig. 4).

### Subcellular localization nature

Subcellular localization analysis was predicted by CELLO and validated by PSORTb, SOSUIGramN, and PSLpred. The subcellular localization of the hypothetical protein was predicted to be a cytoplasmic protein (Table 4). Absent of transmembrane helices predicted by THMM and HMMTOP also emphasizes the result of being a cytoplasmic protein. Also, CCTOP server predicted that the query protein was not a transmembrane protein. All these results summarize the protein as a cytoplasmic one.

### Secondary structure analysis

The SOPMA secondary structure prediction server analysis revealed the proportions of alpha helix, beta turn, extended strand, and the random coil of protein as 31.89%, 9.11%, 18.87%, and 40.13%, respectively (Supplementary Fig. 2).

### Three-dimensional structure analysis

**Fig. 5.**
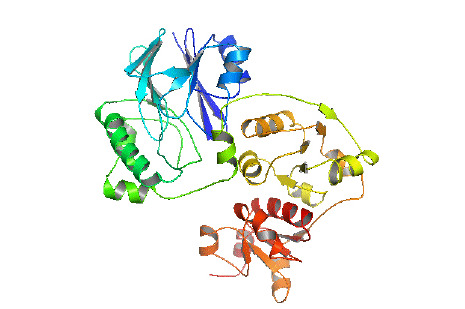
Predicted three-dimensional structure of the hypothetical protein.

**Fig. 6.**
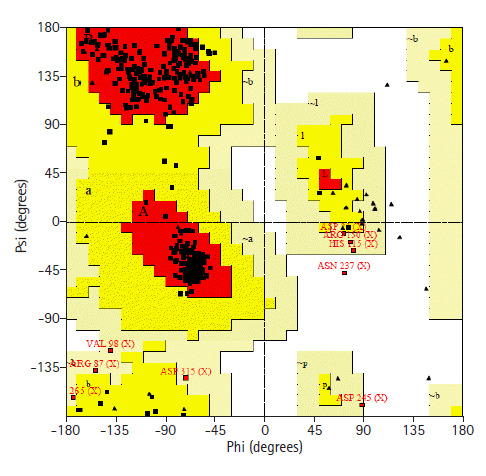
Ramachandran plot of modelled structure validated by PROCHECK program.

Prediction of 3D structure was done by HHPRED server. The server predicted 3D structure of the protein with 100% identity with the highest scoring template (PDB ID: 3TP9_A) (Fig. 5). 3TP9 is the crystal structure of *Alicyclobacillus acidocaldarius* protein with β-lactamase and rhodanese domains. This protein is a homo-dimer which has two chains (chain A and chain B) and the chain A was used as template to build the model. Validation of the predicted three-dimensional model was assessed by PROCHECK through Ramachandran plot analysis, where the distribution of φ and ψ angle in the model within the limits are shown (Table 5, Fig. 6). Residues in the most favored regions covered 90.9%, which is the quality of a valid model. Finally, the established model of 3D structure for the target sequence was verified by structure validation server Verifiy3D and ERRAT. In the Verify3D graph, 92.73% of the residues have averaged 3D-1D score ≥ 0.2 which indicates that the environmental profile of the model is good and the overall quality factor predicted by the ERRAT server was 69.0583 indicates a good model. The 3D structure was later modified by YASARA energy minimization server. The energy calculated before energy minimization was –77,930.2 kJ/mol whereas after energy minimization (through 3 round of steepest descent method), it was changed to far less value of –244,148.6 kJ/Mol making the modeled structure more stable one.

### Protein-protein interaction analysis

**Fig. 7.**
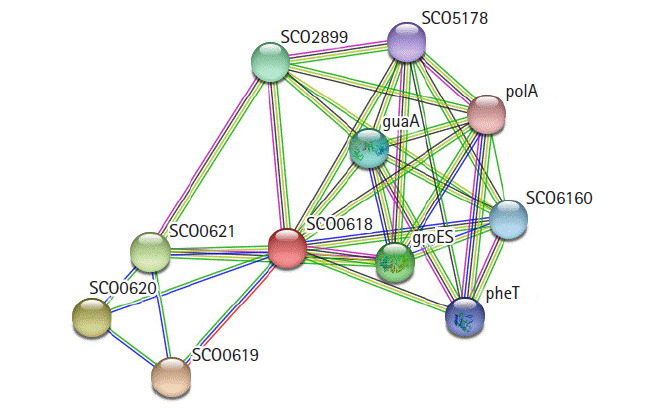
String network analysis of the hypothetical protein, indicates as SCO0618.

STRING 10.0 search was carried out for the identification of possible functional interaction network of the protein [31]. The identified functional partners with scores were; SCO0619 (0.970), SCO0620 (0.743), SCO0621 (0.739), groES (0.568), SCO2899 (0.568), guaA (0.545), SCO6160 (0.520), pheT (0.508), SCO5178 (0.485), polA (0.473). Of them, SCO0619 is a possible membrane protein. The others are two hypothetical proteins, two chaperonins, GMP synthase, multifunctional fusion protein, phenylalanine tRNA ligase β subunit, putative sulfurylase, and DNA polymerase I (Fig. 7).

### Active site of the hypothetical protein

**Fig. 8.**
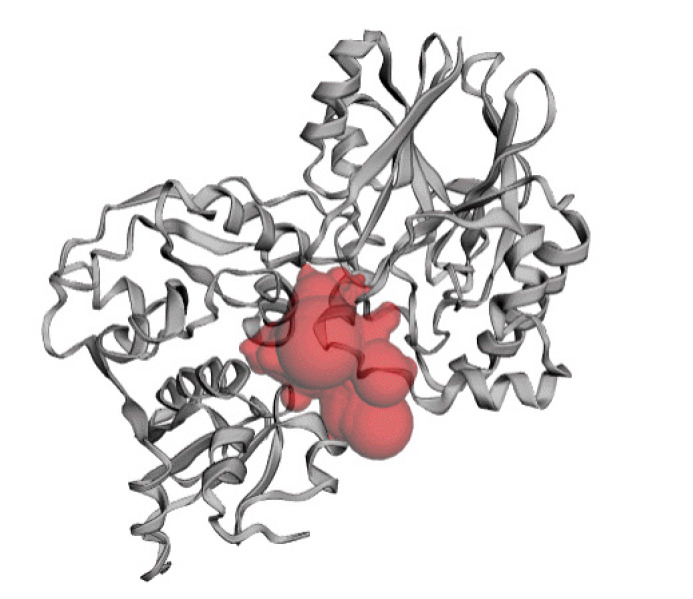
Active site of the hypothetical protein. Here the red sphere indicates the active site of the protein.

The predicted active site of the protein found that 42 amino acids are involved in potent active site (Fig. 8). The best active site was found in areas with 613.075 and a volume of 608.774 amino acids. The amino acid residues in the active site were shown in Supplementary Fig. 3.

### Conclusion

The identification of protein functions is fundamental for the understanding of biological processes. So, this study was aimed to determine the structural and biological function of SCO0618, a hypothetical protein of this bacterium through an in-silico approach. The identified protein revealed several characteristics such as cytoplasmic nature, hydrolytic enzymes containing domain presence, ‘(TIM)-barrel’ fold presence, and hydrolase activity emphasize the significance of this protein. These characters of the hypothetical protein will strengthen basic knowledge on *S. coelicolor*. So, extended in-vitro research has to be carried out to experimentally validate the possibilities shown here and to find out the proteins’ role in biotechnology.

## Figures and Tables

**Table 1.** Tools used for the in-silico characterization of hypothetical protein SCO0618

No.	Server name	Reference	Purpose
1	BLASTp	Johnson et al. (2008) [13]	Similarity search
2	protBLAST	Altschul et al. (1999) [40]	
3	Muscle	Madeira et al. (2019) [14]	Multiple sequence alignment
4	Protparam	Gasteiger et al. (2005) [16]	Physicochemical characterization
5	Psortb	Yu et al. (2010) [18]	
6	PSLpred	Bhasin et al. (2005) [19]	Subcellular localization prediction
7	Cello	Yu et al. (2006) [17]	
8	SOSUIGramN	Imai et al. (2008) [20]	
9	TMHMM	Moller et al. (2001) [21]	Topology prediction
10	HMMTOP	Tusnady and Simon (2001) [22]	
11	CCTOP	Dobson et al. (2015) [23]	
12	Motif	Kanehisa et al. (2002) [25]	Motif discovery
13	Pfam	Finn et al. (2015) [26]	Family relationship identification
14	Superfamily	Wilson et al. (2007) [27]	Superfamily search
15	COILS	Lupas et al. (1991) [28]	Coiled-coil motif identification
16	PFP-FunDSeqE	Shen and Chou (2009) [30]	Fold recognition
17	Interpro	Hunter et al. (2009) [29]	Functional classification
18	STRING	Szklarczyk et al. (2015) [31]	Interaction network analysis
19	PSIPRED	McGuffin et al. (2000) [32]	Secondary structure prediction
20	SOPMA	Geourjon and Deléage (1995) [33]	
21	HHpred	Zimmermann et al. (2018) [34]	Tertiary structure prediction
22	PROCHECK	Laskowski et al. (1993) [36]	
23	Verify3D		Structure verification
24	ERRAT		

**Table 2.** Similar protein obtained from non-redundant UniProt KB/SwissProt sequences

Protein ID	Organism	Protein name	Identity (%)	Score	e-value
WP_011027250.1	*Streptomyces*	MULTISPECIES: MBL fold metallo-hydrolase	100	889	0.0
WP_003978243.1	*Streptomyces*	MULTISPECIES: MBL fold metallo-hydrolase	99.57	886	0.0
WP_121713050.1	*Streptomyces *sp. E5N91	MBL fold metallo-hydrolase	99.13	884	0.0
WP_016325181.1	*Streptomyces lividans*	MBL fold metallo-hydrolase	99.35	883	0.0
WP_093455449.1	Unclassified *Streptomyces*	MULTISPECIES: MBL fold metallo-hydrolase	99.35	883	0.0

**Table 3.** Similar protein obtained from UniProt database

Entry name	Organism	Protein name	Identity (%)	Score	e-value
Q88FF3.1	*Pseudomonas putida* KT2440	Hydroxyacylglutathione hydrolase	32.97	60.1	6e-09
B1JBN3.1	*Pseudomonas putida* W619	Hydroxyacylglutathione hydrolase	31.49	58.2	3e-08
B0KN02.1	*Pseudomonas putida* GB-1	Hydroxyacylglutathione hydrolase	30.77	57.8	3e-08
A5W167.1	*Pseudomonas putida* F1	Hydroxyacylglutathione hydrolase	31.32	55.8	1e-07
D3RPB9.1	*Allochromatium vinosum* DSM 180	Sulfurtransferase	33.33	51.6	3e-07

**Table 4.** Subcellular localization analysis

No.	Analysis	Result
1	CELLO 2.5	Cytoplasmic localization
2	PSORTb	Cytoplasmic localization
3	SOSUIGramN	Cytoplasmic localization
4	PSLpred	Cytoplasmic Protein
5	TMHMM 2.0	No transmembrane helices present
6	HMMTOP	No transmembrane helices present
7	CCTOP	Not transmembrane protein

**Table 5.** Ramachandran plot statistics of the hypothetical protein

Ramachandran plot statistics	No. (%)
Residues in the most favored regions [A, B, L]	351 (90.9)
Residues in the additional allowed regions [a, b, l, p]	26 (6.7)
Residues in the generously allowed regions [a, b, l, p]	8 (2.1)
Residues in the disallowed regions	1 (0.3)
No. of non-glycine and non-proline residues	386
No. of end-residues (excl. Gly and Pro)	2
No. of glycine residues (shown in triangles)	41
No. of proline residues	25
Total No. of residues	454

## Supplementary Materials

Supplementary Fig. 1. Multiple sequence alignment of different homologous protein.

Supplementary Fig. 2. Secondary structure analysis using PSIPRED.

Supplementary Fig. 3. The amino acid residues in the active site (blue color).
