# Supplementary figures and images for: Molecular characterization and functional annotation of a hypothetical protein (SCO0618) of Streptomyces coelicolor A3(2)
Source: Genomics Inform. 2020 Sep 21;18(3):e28. doi: 10.5808/GI.2020.18.3.e28 (PMC7560446; doi:10.5808/GI.2020.18.3.e28)

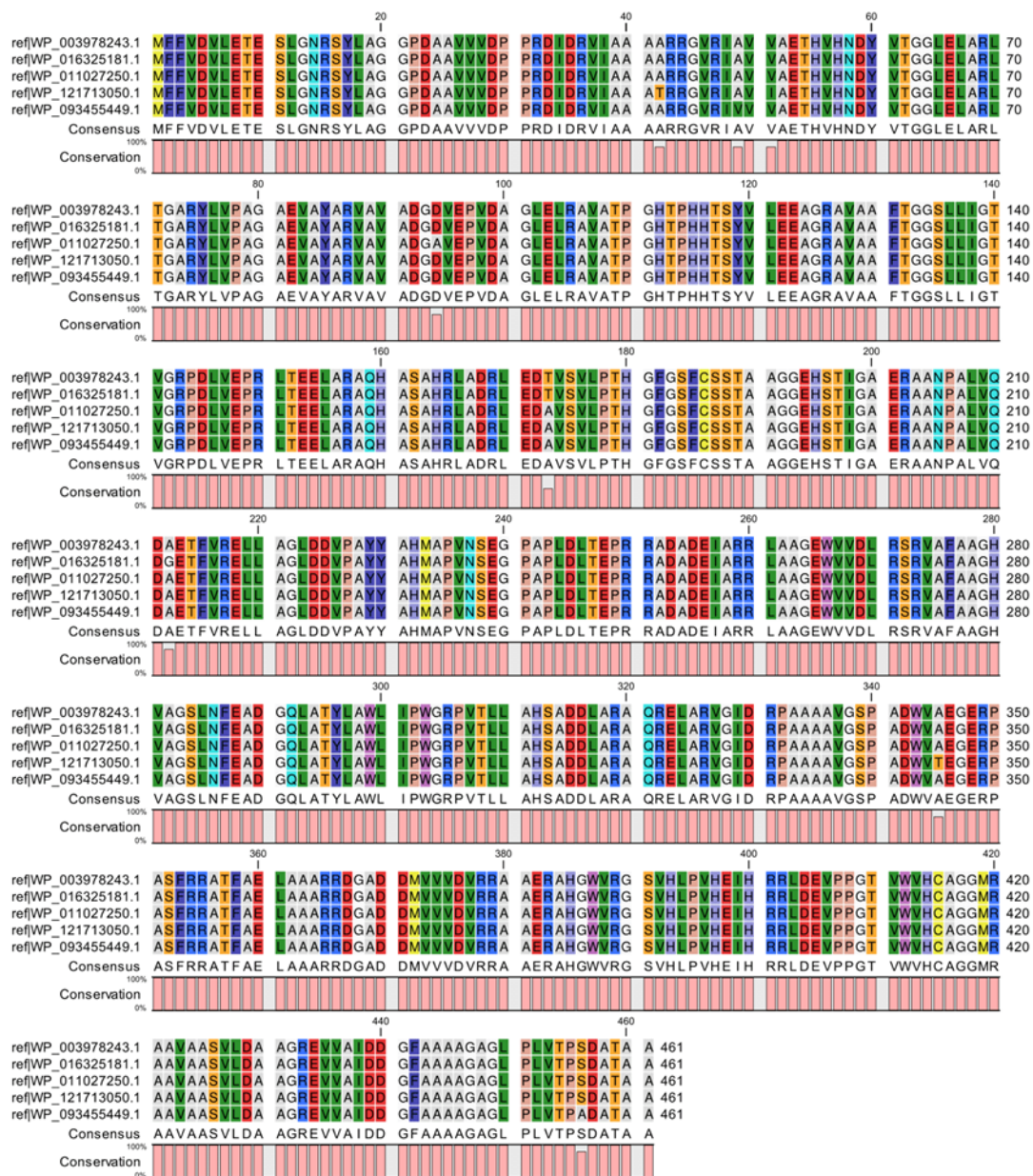

**Supplementary Fig. 1:** Multiple sequence alignment of different homologous protein.

Supplement: Supplementary Fig. 1. — Multiple sequence alignment of different homologous protein. [file gi-2020-18-3-e28-suppl1.pdf]
