# Supplementary material for: Molecular characterization and functional annotation of a hypothetical protein (SCO0618) of Streptomyces coelicolor A3(2)
Source: Genomics Inform. 2020 Sep 21;18(3):e28. doi: 10.5808/GI.2020.18.3.e28 (PMC7560446; doi:10.5808/GI.2020.18.3.e28)
Supplement: Supplementary Fig. 3. — The amino acid residues in the active site (blue color). [file gi-2020-18-3-e28-suppl3.pdf]

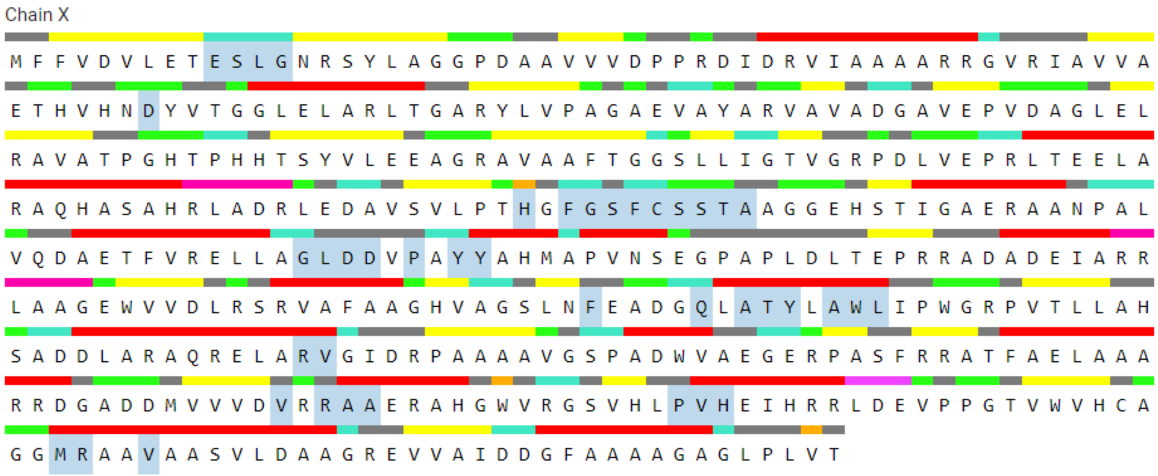

Supplementary Fig. 3. The amino acid residues in the active site (blue color).
