# Supplementary material for: Molecular characterization and functional annotation of a hypothetical protein (SCO0618) of Streptomyces coelicolor A3(2)
Source: Genomics Inform. 2020 Sep 21;18(3):e28. doi: 10.5808/GI.2020.18.3.e28 (PMC7560446; doi:10.5808/GI.2020.18.3.e28)
Supplement: Supplementary Fig. 2. — Secondary structure analysis using PSIPRED. [file gi-2020-18-3-e28-suppl2.pdf]

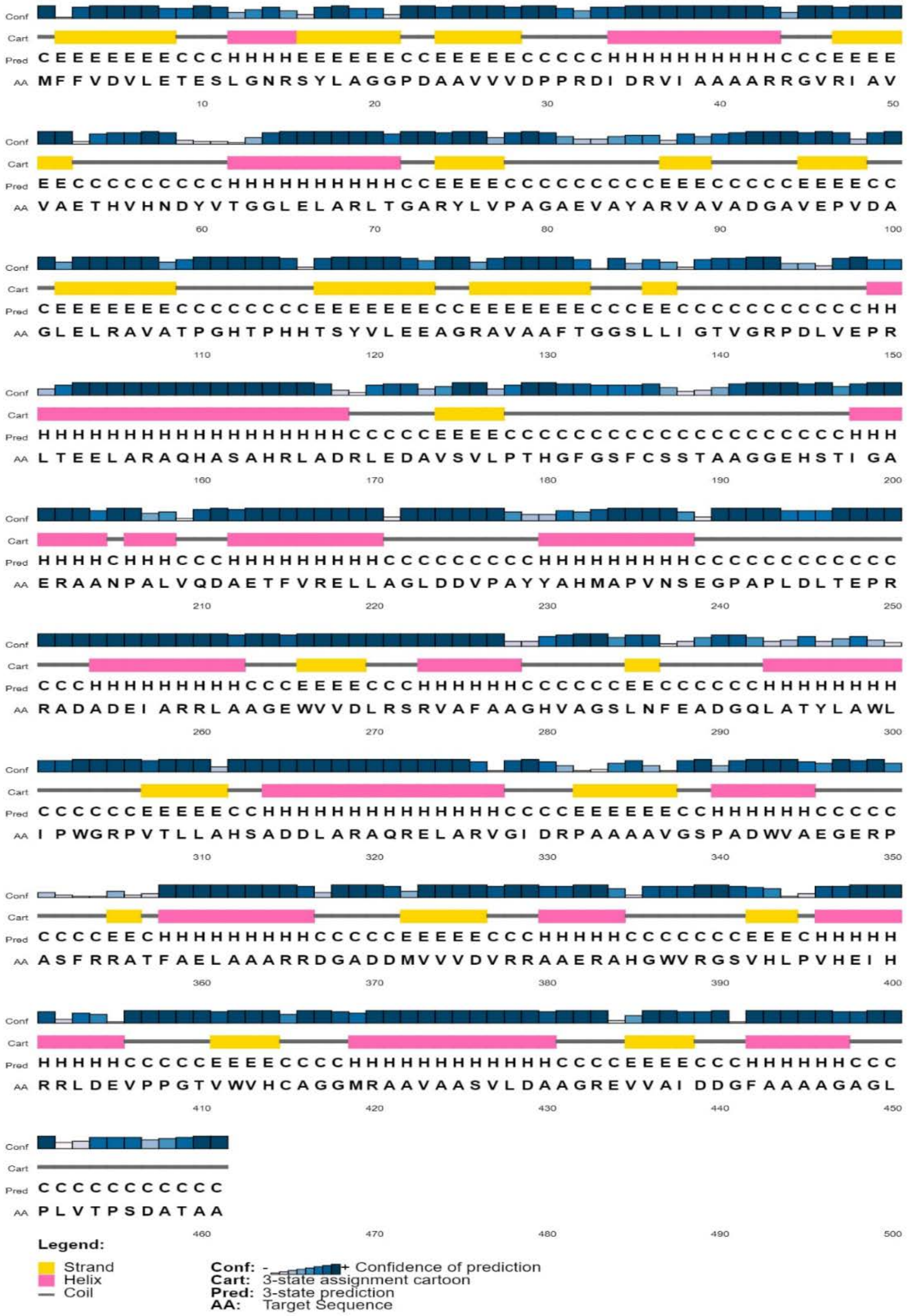

Supplementary Fig. 2. Secondary structure analysis using PSIPRED.
